# Hepatic but Not CNS-Expressed Human C-Reactive Protein Inhibits Experimental Autoimmune Encephalomyelitis in Transgenic Mice

**DOI:** 10.1155/2015/640171

**Published:** 2015-09-03

**Authors:** Tyler T Wright, Rachel V. Jimenez, Todd E. Morgan, Namrata Bali, Xiaogang Hou, Mark A. McCrory, Caleb E. Finch, Alexander J. Szalai

**Affiliations:** ^1^Department of Medicine, The University of Alabama Birmingham, Birmingham, AL 35294, USA; ^2^Davis School of Gerontology and Department of Biological Sciences, The University of Southern California, Los Angeles, CA 90089, USA; ^3^Department of Pathology, The University of Alabama Birmingham, Birmingham, AL 35294, USA

## Abstract

We recently demonstrated that human C-reactive protein (CRP), expressed hepatically in transgenic mice (CRPtg), improved the outcome of experimental autoimmune encephalomyelitis (EAE), a murine model of multiple sclerosis (MS). The liver is the primary site of CRP synthesis in humans and in CRPtg mice but is also expressed by both at low levels in the CNS. To determine if CNS expression of human CRP is sufficient to impact EAE, we generated neuronal CRP transgenic mice (nCRPtg) wherein human CRP expression is driven by the neuron-specific Ca^2+^/calmodulin-dependent protein kinase II*α* (CaMKII*α*) gene promoter. We found that hepatically expressed/blood-borne CRP, but not CNS expressed CRP, lessened EAE severity. These outcomes indicate that the protective actions of human CRP in EAE are manifested in the periphery and not in the CNS and reveal a previously unappreciated site specificity for the beneficial actions of CRP in CNS disease.

## 1. Introduction

C-reactive protein (CRP) is a blood-borne acute phase reactant produced primarily by hepatocytes [1]. It binds ligands expressed on bacteria and damaged tissues and subsequently promotes inflammatory and immune processes via activation or inhibition of complement or by engagement of various Fc receptors [2, 3]. Growing evidence indicates that blood-borne CRP can cross the blood-brain and blood-spinal cord barriers, and thus CRP can be found in the cerebrospinal fluid (CSF) [4, 5] and is deposited in the diseased CNS [6–8]. The source of CRP might also be local however, as CRP production by multiple CNS-resident cells including neurons, microglia, and astrocytes has been reported [9–11]. Regardless of its origin (hepatic versus local), the presence of CRP in the CNS is associated with numerous diseases including Alzheimer's disease [8, 9, 12], amyotrophic lateral sclerosis [13], and multiple sclerosis (MS) [12, 14].

Our laboratory has used transgenic mice expressing human CRP in the liver (CRPtg) [15] to demonstrate that blood-borne human CRP ameliorates experimental autoimmune encephalomyelitis (EAE), a rodent model of MS [16]. We observed delayed onset and milder clinical symptoms of EAE in CRPtg compared to wildtype mice, an outcome we attribute to human CRP-mediated suppression of encephalitogenic T cells [16]. This scenario is consistent with the protective effect of hepatically derived/blood-borne human CRP that we have documented in other mouse models of autoimmunity [17]. Additional EAE studies showed that human CRP's ability to ameliorate disease in CRPtg depends on its ability to engage the inhibitory type IIB Fc gamma receptor (Fc*γ*RIIB) [18], a finding which may partially explain the tonic suppressive effect of CRP on T cell-driven autoimmunity [16, 17]. It remains unknown, however, whether human CRP exerts its protective effect on EAE in the periphery or in the CNS. The present study was performed to directly address this question.

We generated mice wherein expression of a human* CRP* transgene is directed by the Ca^2+^/calmodulin-dependent protein kinase II*α* (CaMKII*α*) gene promoter [19]. CaMKII*α* is constitutively expressed by neurons in the brain and spinal cord [19–23]. We demonstrate that, in these neuronal CRPtg mice (nCRPtg), human* CRP* mRNA expression and protein production are restricted to the CNS and that the presence of CRP* per se* does not alter the inflammatory state of the CNS parenchyma. Unlike blood-borne human CRP of hepatic origin [16, 18], CSF-borne CRP of CNS origin did not attenuate EAE. This finding strongly suggests that CRP's beneficial actions in EAE likely are manifested in the periphery and reveals a previously unexpected site-specificity to the influence of CRP in autoimmune disease of the CNS.

## 2. Materials and Methods

### 2.1. Generation of nCRPtg Mice

**Figure 1:**
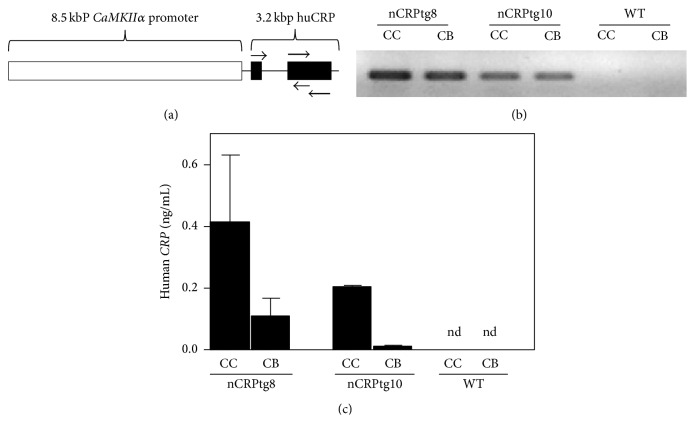
The nCRPtg construct and generation of nCRPtg mice. (a) The transgene comprises the protein coding region of the human* CRP* gene (huCRP; black boxes) fused to the mouse Ca^2+^/calmodulin-dependent protein kinase *α* (*CaMKIIα*) gene promoter (open box). Small arrows indicate the approximate positions and directions of elongation of the intron-flanking sense and antisense primers used to ensure proper* CRP* mRNA processing, and the large arrows show the location of primers used to genotype mice. Horizontal dimension is not to scale. (b) Agarose gel electrophoresis of amplification products generated by human* CRP* specific qRT-PCR. mRNA for qRT-PCR was isolated from the cerebral cortex (CC) and cerebellum (CB) and qRT-PCR was performed using primers that flanked the* CRP* intron (small arrows in (a)). The size of the resulting reaction product (~290 bp) indicates excision of the intron. (c) Enzyme linked immunosorbent assay of cerebral (CC) and cerebellar (CB) homogenates from the same mice shown in (b). nd, not detected. WT, wildtype mouse.

To target expression of human CRP to the CNS we utilized a mouse* CaMKIIα* gene promoter construct previously employed to drive neuronal expression of other transgenes [19, 21–23]. The* CaMKIIα* promoter clone was a generous gift of Mark Mayford (The Scripps Research Institute, La Jolla, CA) and has been fully described elsewhere [19]. Briefly, a 3.2 kbp cDNA insert encoding the human* CRP* gene was digested by NotI and ligated into the unique* NotI* site of pMM403, immediately upstream of the 8.5 kbp* CaMKIIα* promoter (Figure 1(a)). The orientation of the* CRP* insert and the integrity of the resulting* CaMKIIα-CRP* construct (Figure 1(a)) were checked by linearization of the plasmid via HindIII digestion followed by direct DNA sequencing. Expression of* CaMKIIα-CRP* mRNA was verified by transient transfection of cultured mouse neuronal N2a cells (data not shown), and transgenic mice were subsequently made in the USC Cancer Center Transgenic Core Facility by injecting the linearized* CaMKIIα-CRP* construct directly into C57BL/6 pronuclei, followed by zygote transfer into pseudopregnant females. Genomic DNA isolated from the resultant pups was screened by polymerase chain reaction (PCR) to detect the transgene (Figure 1(a)). Two rounds of injections yielded five nCRPtg positive mice. CRPtg mice, also on the C57BL/6 background, have been described in detail elsewhere [15]. In CRPtg, human CRP is expressed primarily in the liver and acts as an acute phase reactant, reaching blood levels comparable to those observed in humans with inflammatory disease (up to 500 *μ*g/mL) [15]. All mice were fed a standard mouse pellet diet (Ralston Purina Diet)* ad libitum* and maintained under standard conditions at constant humidity (60 ± 5%) and temperature (24 ± 1°C) with a 12-hour light cycle (6 a.m. to 6 p.m.). All protocols were approved by the Institutional Animal Care and Use Committee at the University of Alabama at Birmingham and were consistent with the* Guide for the Care and USE of Laboratory Animals* published by the National Institutes of Health (NIH publication 96-01, revised 1996).

### 2.2. *In Situ* Hybridization and Immunofluorescence

Anesthetized mice were perfused with cold phosphate-buffered saline (PBS) via transcardial perfusion. A small incision was made in the right atrium, a sterile syringe was inserted into the left ventricle, and PBS was slowly injected until all organs were fully perfused. Brains, spinal cords, and livers were then removed and processed. Frozen sections (10–16 *μ*m) were used for* in situ* hybridization and immunofluorescence studies. Sense and antisense digoxigenin-labeled riboprobes specific for human* CRP* mRNA were used for hybridization [17], followed by sequential washing and immunodetection using an antibody conjugated to alkaline phosphatase. Hybridization signals were developed overnight using nitro blue tetrazolium chloride/5-bromo-4-chloro-3-indolyl phosphate (NBT/BCIP) substrate (Roche) and images were captured by standard light microscopy. For immunofluorescence, frozen sections were stained with a monoclonal antibody against human CRP directly conjugated to fluorescein isothiocyanate (FITC) (HyTest, Turku, Finland; mAb 4C28F) and counterstained with a monoclonal antibody against the nuclear antigen NeuN (a marker of neurons) directly conjugated to biotin (Millipore, Billerica, MA; mAb377). The anti-NeuN antibody was detected using a streptavidin-Texas Red conjugate (Rockland Immunochemicals, Gilbertsville, PA; catalog number S000-09). Nuclei were stained using 4′,6-diamidino-2-phenylindole (DAPI) (IHC World, Woodstock, MA). Fluorescence micrographs were captured using a Leica DMRB microscope at 40x magnification.

### 2.3. Quantitative Reverse Transcription PCR (qRT-PCR)

To measure human* CRP* expression total RNA was extracted from perfused isolated organs using TRIZOL according to the manufacturer's instructions (Invitrogen, Grand Island, NY). RNA was then treated with DNaseI (Ambion, Grand Island, NY) and cDNA was synthesized using the iScript kit (Bio-Rad, Hercules, CA). SYBR Green Mastermix (Bio-Rad) and human* CRP* primers were used to amplify cDNA on a CFX96 thermocycler (Bio-Rad). To assess mRNA processing we used* CRP* primers that annealed to exon 1 (sense: 5′-CTCTCATGCTTTTGGCCAGAC-3′) and to exon 2 (antisense: 5′-CTACTGTGACTTCAGGAACCTC-3′) (Figure 1(a), small arrows), yielding an amplicon of 576 bp if the intron was present or 288 bp if the intron was absent (Figure 1(b)). Otherwise we used primers that amplified a 209 bp stretch of exon 2 (sense: 5′-TTTACAGTGGGTGGGTCTGAA-3′ and antisense: 5′-CCACCGAAGGAATCCTG-3′) (large arrows in Figure 1(a)). All* CRP* qRT-PCR amplification reactions were run using the following conditions: initial denaturation at 95°C for 5 min, followed by 50 cycles of amplification (95°C for 10 sec, 55°C for 30 sec, and 72°C for 30 sec) and a final elongation at 65°C for 30 sec. To ensure purity, melt curve analysis was done starting at 95°C with a temperature decrease of 0.5°C at each step. To measure expression of neuronal nitric oxide synthase (*nNOS*), inducible nitric oxide synthase (*iNOS*), endothelial nitric oxide synthase (*eNOS*), glial acidic fibrillary protein (*GFAP*), and* CD68*, we used similar protocols, differing only in primer sequences and annealing temperatures. The primer sequences and annealing temperatures are as follows:* nNOS* (sense: 5′-GGGCAAACAGTCTCCTACCA-3′ and antisense: 5′-AGGGTGTCAGTGAGGACCAC-3′, annealing temperature of 60°C);* iNOS* (sense: 5′-TGCATGGACCAGTATAAGCAAGC-3′ and antisense: 5′-GCTTCTGGTCGATGTCATGCAA-3′, annealing temperature of 60°C);* eNOS* (sense: 5′-TGTCTGCGGCGATGTCACTATG-3′ and antisense: 5′-CGAAAATGTCCTCGTGGTAGCG-3′, annealing temperature of 60°C);* GFAP* (sense: 5′-GCAGAAGCTCCAAGATGAAAC-3′ and antisense: 5′-CCTTTCTCTCCAAATCCACAC-3′, annealing temperature of 54°C);* CD68* (sense 5′-GCACAGTGGACATTCATGGC-3′ and antisense: 5′CGGTTGCAAGAGAAACATGG-3′, annealing temperature of 51.3°C). For each gene of interest mRNA expression was normalized to expression of glyceraldehyde 3-phosphate dehydrogenase (*GAPDH*), amplified using the following primers: sense: (5′- ATTCTTCCACCTTTGATGC-3′) and antisense: (5′- TGGTCCAGGGTTTCTTACT-3′). All expression data are expressed using the ΔCt method.

### 2.4. CSF and Blood Collection

CSF was collected using a modification of an established method [24]. Briefly, under deep anesthesia an incision was made from the occipital bone down the back of the neck, separating the musculature and exposing the meninges overlying the cisterna magna. A small capillary tube was inserted gently between the occipital protuberance and the spine of the atlas, thus penetrating the atlantooccipital membrane. CSF was slowly collected by allowing internal luminal pressure to fill the capillary; yields of 10–20 *μ*L of clear fluid were typically obtained with no sign of blood contamination. CSF samples were stored at 4°C and analyzed within 24 hours. Heparinized blood was collected by submandibular cheek punch [25] and the plasma was stored at −20°C until analysis less than one week later. In some instances, CSF and blood were collected from mice 24 hours following i.p. administration of 25 *μ*g of bacterial lipopolysaccharide (*E. coli* 055:B5, Sigma-Aldrich). Blood, CSF, and brain homogenate CRP levels were determined by a human CRP specific ELISA.

### 2.5. Induction of EAE

Myelin oligodendrocyte protein (MOG) peptide (MOG_35–55_) was used to immunize mice as previously described [16, 18]. On day 0, mice received a subcutaneous injection of 150 *μ*g MOG_35–55_ emulsified in incomplete Freund's adjuvant containing 500 *μ*g heat-killed* Mycobacterium tuberculosis* (Difco, Detroit, MI). Also on day 0 and again on day 2, mice received an intraperitoneal injection of 200 ng pertussis toxin (List Biological Laboratories, Campbell, CA). Development of EAE symptoms was monitored daily using a clinical scale ranging from 0 to 6 as follows: 0, asymptomatic; 1, loss of tail tone; 2, flaccid tail; 3, incomplete paralysis of one or both hind limbs; 4, complete hind limb paralysis; 5, moribund (in which case animals were humanely euthanized); 6, dead. Mice were observed for at least 30 days. All immunized mice developed EAE, and with the day of onset considered to be the first of two consecutive days the clinical score reached or eclipsed 2. The maximum clinical score achieved by each animal during the 30-day observation period was averaged for each genotype and used to estimate disease severity, and for each genotype the daily average clinical score was plotted. The cumulative disease index (CDI) was determined by summation of clinical scores from the day of disease onset to day 30.

### 2.6. Statistical Analysis

All statistics are presented as means with associated standard errors. Group differences were determined by one-way analysis of variance (ANOVA) and post hoc Tukey's multiple comparison tests. In all cases, a value of *p* < 0.05 was considered significant.

## 3. Results and Discussion

**Figure 2:**
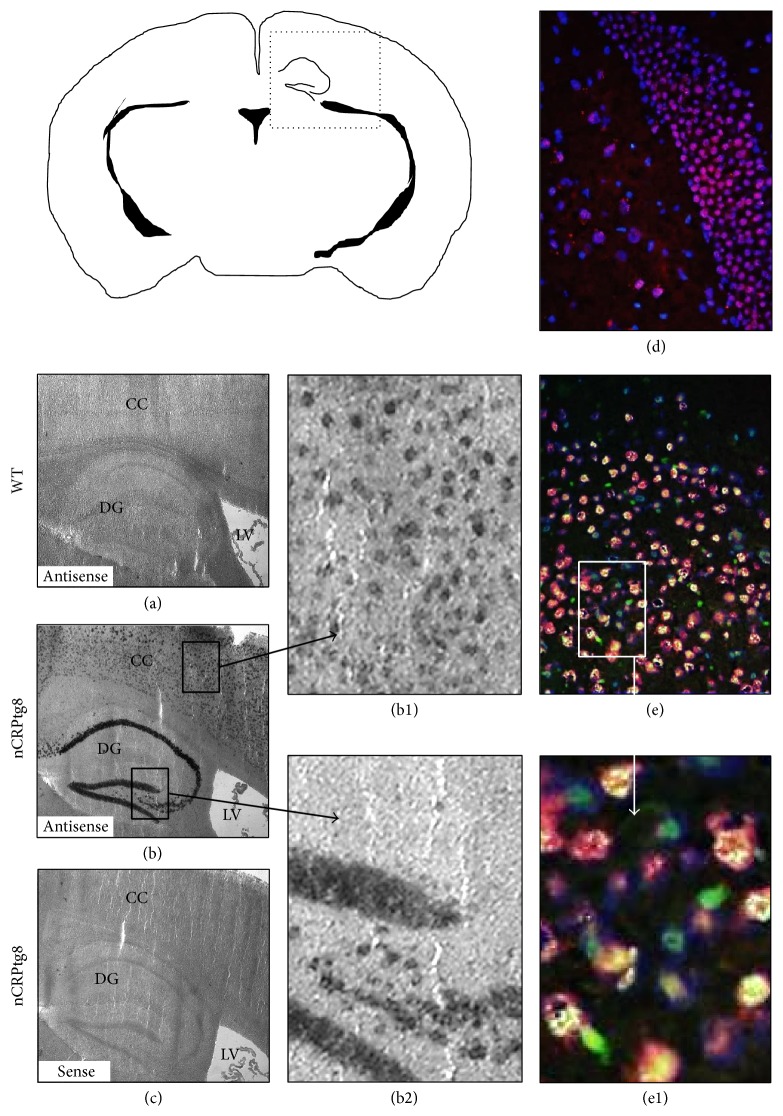
CNS-restricted expression of the* CaMKIIα*-driven human* CRP* transgene and CRP production in the CNS of nCRPtg mice. ((a)–(c)) Thin coronal sections of brains from a wildtype (WT, (a)) and a nCRPtg mouse ((b) and (c)) were incubated with a digoxigenin-labelled antisense probe to localize human* CRP* mRNA or with a similarly labeled sense probe to reveal nonspecific hybridization. Note the strong hybridization signals generated by the antisense probe in the cerebral cortex (CC) and dentate gyrus (DG) of nCRPtg mice ((b); (b1) and (b2) are tenfold magnifications of the areas indicated). LV = lateral ventricle. ((d)-(e)) Frozen sections of wild type (d) and nCRPtg brains ((e); (e1) is a tenfold magnification of the indicated area) were incubated with a FITC-conjugated anti-human CRP antibody (green) and a Texas Red-conjugated anti-NeuN antibody (red). An orange-yellow color indicates colocalization of the FITC and Texas Red signals. DAPI (blue) was also used to stain cell nuclei. The dashed box in the cartoon shows the approximate position and orientation of the thin sections shown in (a)–(e).

**Figure 3:**
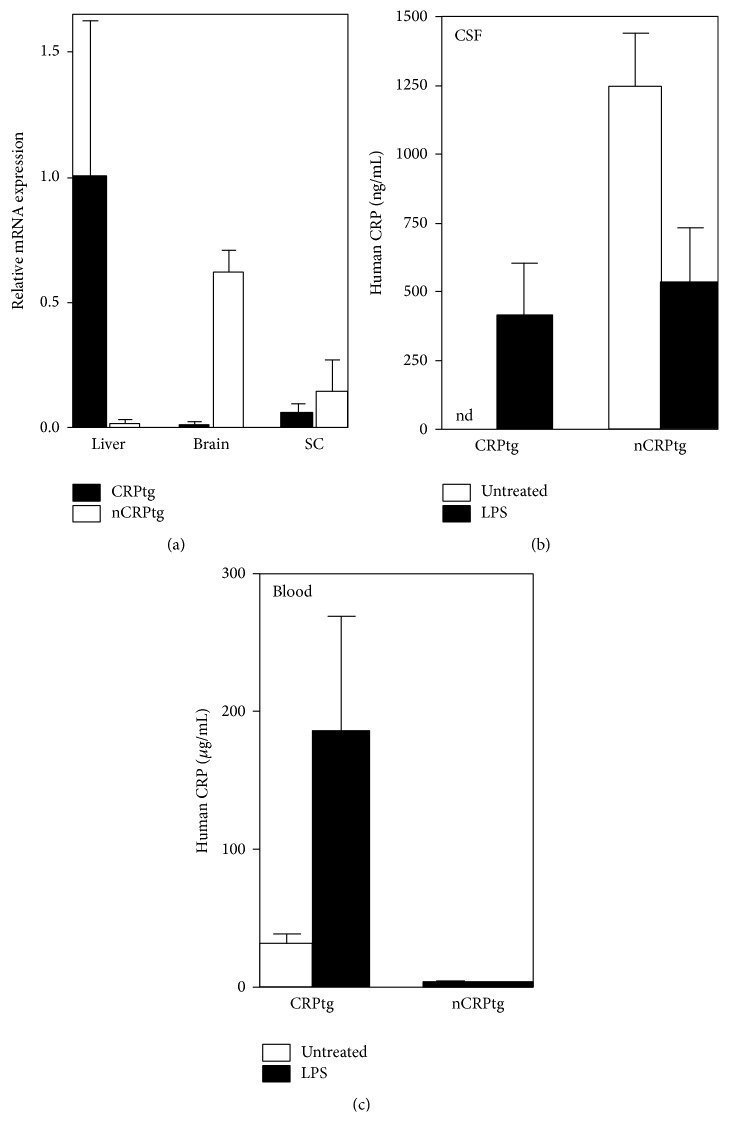
Human CRP is expressed exclusively in the CNS in nCRPtg mice. (a) qRT-PCR of mRNA isolated from livers, brains, and spinal cords (SC) of CRPtg (black bars) and nCRPtg mice (open bars). The data shown are from *n* = 3 mice of each genotype and are normalized to baseline expression of* CRP* by CRPtg livers. Robust human* CRP* mRNA is detectable in the CNS of nCRPtg and in the livers of CRPtg. (b) Human CRP protein levels in the CSF collected from CRPtg and nCRPtg mice, injected with LPS, or left untreated (*n* = 3 mice per group). Human CRP was detected in the CSF of nCRPtg but was not detected (nd) in the CSF of CRPtg. (c) as in (b), but showing blood levels of human CRP, only detectable in the plasma of CRPtg.

Two of the original five transgenic mice (nCRPtg8 and nCRPtg10) were sacrificed and used to confirm that* CRP* mRNA was expressed and appropriately processed* in vivo* (Figure 1(b)) and that CRP protein was present in the CNS (Figure 1(c)).* In situ* hybridization using digoxigenin-labelled riboprobes and immunofluorescence microscopy utilizing anti-human CRP in combination with anti-NeuN antibodies confirmed that human* CRP* mRNA (Figure 2, (b), (b1), and (b2)) and CRP protein (Figure 2, (e) and (e1)) were both localized to neurons within the CNS. The remaining nCRPtg mice were mated to wildtype C57BL/6, which confirmed germline transmission of the transgene for nCRPtg13. nCRPtg13 thus served as the founder and was mated to wildtype C57BL/6 mice to produce animals for all subsequent studies. Descendants of nCRPtg13 robustly express human* CRP* mRNA in their brains and spinal cords, but not in their livers (Figure 3(a)). More importantly, human CRP protein was detected in the CSF of nCRPtg (Figure 3(b)) but not in the blood, even after LPS injection (Figures 3(b) and 3(c)). As initially reported [15], in CRPtg we observed robust human* CRP* expression in the liver (Figure 3(a)) and a large quantity of CRP protein in the blood, especially after LPS injection (Figure 3(c)). These results are consistent with the CNS as the exclusive source of human CRP in nCRPtg. No embryonic lethality, gender specificity, or unusual phenotype related to neuronal CRP has been observed to date.

**Figure 4:**
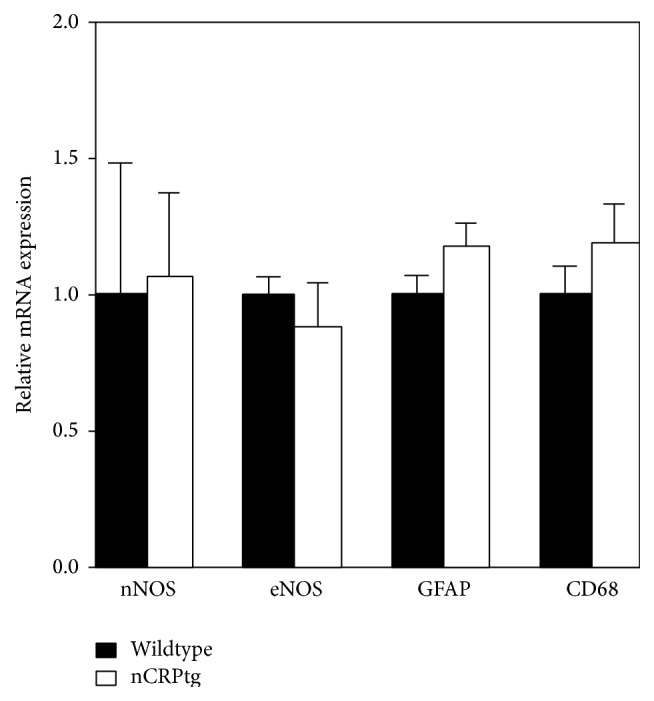
CNS-expressed human CRP does not upregulate the expression of inflammatory biomarkers in the CNS. qRT-PCR of mRNA isolated from the brains of wildtype and nCRPtg mice, *n* = 3 per genotype. Human CRP expression in nCRPtg brains does not influence the expression of endothelial or neuronal nitric oxide synthases (eNOS and nNOS, resp.), GFAP, or CD68. Inducible nitric oxide synthase (iNOS) was not detected in either genotype. Expression of each gene is normalized to expression in wildtype mice.

**Figure 5:**
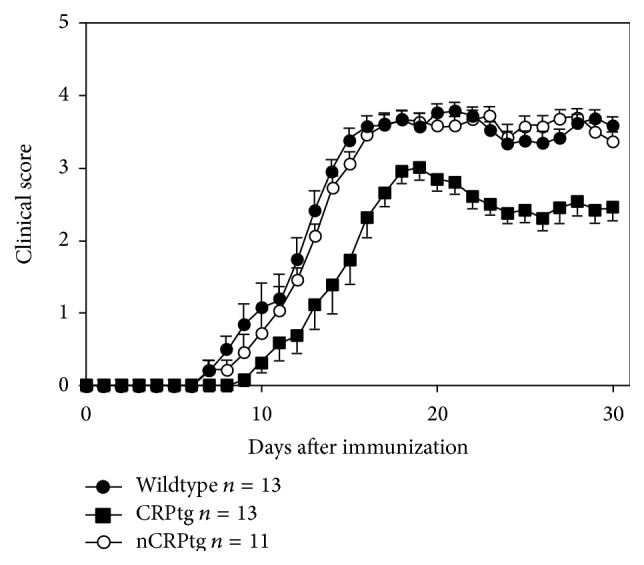
CRPtg, but not nCRPtg mice, are resistant to EAE. Wildtype, CRPtg, and nCRPtg mice were immunized with MOG peptide and symptoms of EAE were monitored over the course of 30 days. EAE onset was significantly delayed and its symptoms ameliorated in CRPtg, but not in nCRPtg (see Table 1).

To gauge whether neuronally expressed CRP altered the inflammatory environment of the CNS, we measured baseline expression of several different biomarkers of CNS inflammation. None of these markers were upregulated in the CNS of nCRPtg (Figure 4). Having verified that human* CRP* expression and CRP protein are restricted to the CNS in nCRPtg and that the presence of CRP in the CNS is not inflammatory, we tested whether CNS-expressed CRP was sufficient to influence the outcome of MOG peptide-induced EAE. As we reported previously [16, 18], we found that EAE onset was significantly delayed and EAE severity was significantly reduced in CRPtg compared to wildtype, but neuronal expression of CRP had no such impact (Figure 5 and Table 1).

Expression of human CRP in the CNS has been documented by several groups [9–11] and while local expression is low relative to that seen in the liver, it may nevertheless have important physiological and/or psychological consequences [26–28]. In this paper, we report the generation of a new transgenic mouse wherein human* CRP* is expressed by a neuron-targeting transgene. To our knowledge, this is the first human CRP transgenic animal wherein human CRP is expressed by cells other than those in the liver and the first model wherein the human CRP protein production is restricted to the CNS. Using these mice we provide important new evidence that CRP expression in the CNS* per se* does not alter the environment of the CNS, yet unlike hepatically expressed human CRP, which delays the onset and decreases the clinical severity of EAE [16, 18], CNS-specific expression of human CRP is not sufficient to modify the course of EAE.

## 4. Conclusion

These new findings strongly support our earlier contention that blood-borne/hepatically synthesized human CRP exerts its protective actions against EAE outside the blood-brain-barrier of CRPtg [16, 18, 29]. Our previous studies indicate that this beneficial effect is most likely the result of suppression of the encephalitogenic T cell response, which is achieved either indirectly by CRP acting on one or more Fc*γ*RIIB expressing antigen-presenting cells [16], by directly binding to naïve T cells [29], or through the combination of both mechanisms. Thus when expression of human CRP is restricted to the CNS, as is the case in nCRPtg, human CRP likely has little opportunity to impact the generation of encephalitogenic T cells in the periphery, and thus it cannot act to delay EAE onset or ameliorate its symptoms. This finding provides compelling new evidence that the biological impact of CRP is site specific.

## Figures and Tables

**Table 1:** The outcome of EAE in wildtype and human CRP transgenic mice^a^.

Strain (*n*)	Disease onset, days mean (sem)	CDI mean (sem)	Maximum score mean (sem)
Wildtype (13)	11.46 (.65)	61.65 (1.68)	3.88 (.08)
CRPtg (13)	14.77 (.61)^b^	44.58 (3.38)^c^	3.29 (.16)^ns^
nCRPtg (11)	12.09 (.51)^ns^	65.77 (1.61)^ns^	3.95 (.05)^ns^

ANOVA	*p* = 0.0006	*p* < 0.0001	*p* = 0.03

^a^Mice were immunized with MOG peptide and the day of onset of EAE as well as its incidence and severity were determined as described in Section 2. The data are pooled from two experiments.

^b^ *p* < 0.05, Neuman-Kuel's multiple comparison test.

^c^ *p* < 0.05, Tukey's multiple comparison test.

^ns^not significant.
